# Indents

**Published:** 1906-08-15

**Authors:** 


### INDENTS.

☞ **Brief Articles of Technical or Scientific Value will receive notice and comment. Contributions invited.**

**Vice Versa.** The old theory that a bad stomach affects the teeth is rapidly giving place to the new theory that a bad stomach, instead of causing bad teeth, is caused by bad teeth.—*Dental Era.*

**To Soften Calculus.** Before removing salivary or serumal calculous it will be found advantageous to apply tincture of iodine. A few treatments of this will tend to disintegrate the deposit and thus facilitate its removal.—E. M. S. Fernandez, Chicago.

**Sterilizing Instruments.** I use equal parts of alcohol and formalin for sterilizing all sharp instruments. Il left in this solution for five or ten minutes, they will be perfectly sterile and not harmed by corrosion. Other instruments I first scrub with sapolio and then boil in soft water with a little soda it it.—W. H. Whitslar, in *Summary.*

**Died from Effects of Chloroform.** Mrs. Anton Williamson died in the chair of a dentist in Osage, Ia., April 4, after chloroform had been administered for extraction of teeth. One tooth had been extracted when the heart ceased beating. Upon reviving she complained of pain, and again losing consciousness, died before a physician could reach her.

**Care of Tooth Brush.** The tooth brush may be kept sterile by taking a large size test tube and constructing it about one inch from the bottom, providing it with a rubber cork as a stopper and placing a little formalin tablet or formaldehyde in the lower chamber and the brush in the upper chamber. It will then become sterile without injury to the brush.—Dr. T. H. Hardgrove, *Deview.*

**Trinity of Anesthetics.** Were I to practice either dentistry or surgery, or both, for another fifty years, I should strive to possess in perfection a knowledge of the properties of cocain, nitrous oxide and chloroform, using them either singly or in combinations, as the case might require. Each should, however, be looked upon with both, adoration and fear.—A. C. Hewitt, in *Dental Summary.*

**Flux for Soft Soldering.** Pieces of zinc are dissolved in hydrochloric acid until the acid is saturated. The resultant solution of zinc chloride is mixed with an equal amount of a mixture consisting of acqua ammonia and alcohol. After standing a few days the solution is filtered and ready for use. The so-called Miller's soldering fluid consists of a solution of phosphoric acid in eight parts of water, to which one part each of lactic acid and glycerine has been added.—The *Dental Era.*

**Controlling Alveolar Hemorrhage.** Soften gutta-percha and press it into the alveolar socket with a considerable ex-l cess. Have the patient bite into it to occlusion, and force the excess against the buccal and lingual surfaces. Take out and cool and trim away such portions as are not needed and make smooth. Replace and let the patient hold it in place by closing the teeth. It makes an effective mechanical plug, which may be of use in retaining medicinal agents.—Dr. E. A. Bogue, in the *Journal.*

**Putrescent Canals.** I have been using dentalone with the best of results in putrescent canals, in this way: Mix a little Dentalone with iodoform. The old way was to mix iodoform with beechwood creosote, which does the work fairly well, but gives off a very disagreeable odor to the dentist and his patient. With the use of dentalone, the odor is greatly modified. I think it does the work better than creosote and I have set the creosote bottle aside and dentalone has taken its place for good. I would suggest that my brother practitioners try dentalone for results.—W. P. Brinton, D. D. S., Bradford, Pa.

**Thymophene.** Equal parts by weight of carbolic acid and thymol crystals rubbed together in a mortar or combined by gentle heat in a test-tube, result in a fluid of oily consistency, similar in characteristics to campho-phenique. It is not escharotic and will not coagulate the cuticle when applied topically. It has high germicidal efficiency. It makes an ideal dressing for root canals, taking the place of campho-phenique and the essential oils, because of its diffusibility, non-irritant, but sedative reaction. A large clinical use warrants a strong recommendation to the profession.—E. C. Kirk, in *Cosmos*.

**Soldering Pliers.** Take a pair of stiff soldering tweezers about six inches long and file a flat notch about an inch long on the inside of each point. Then take a piece of heavy iridio-platinum wire about two inches long and roll it flat and broad enough at one end to notch the width of the tweezer points. Drill two or three holes through each tweezer point and the flat ends of the iridio-platinum wire to match. Then with some soft steel wire rivet the iridio-platinum points to the tweezer points. You can then shape the points to your liking, and you will have a handy pair of soldering tweezers that will not corrode in acid or burn in any flame.—*Dental Record*.

**Capping Pulps** Remove all decayed dentine that is practicable without injuring the pulp tissue then wash the cavity with luke-warm chloroform water and dry with absorbant cotton. Then wipe the cavity with a pledget of cotton saturated with the following solution: Dissolve fifteen grains of thymol in one dram each, of oil of cloves and chloroform. Then mix oxide of zinc and eugenol to the consistance of soft putty and cover the exposure and partly fill the cavity with it. Over this place a temporary protecting filling for several days. After sufficient time for testing if the tooth is comfortable, remove the dressing and refill with a paste of zinc oxide and eugenol paste and cover with a cement protecting filling.—F. H. Aujubalt, in *Cosmos*.

**How a Dental College looks to Some Practitioners.** My idea of the dental college is that there are too many—that the dental college is a machine, grinding out young men and young women to fill teeth of course, the ambition to be a professional man or a professional woman is all right, but it is the type of man who pays attention to his studies and after he graduates pays proper attention to his work, from whom we get the best work. It is the class of men who rub the plaster off the walls in their effort to squeeze through who go into the dental parlors. I know one young man a graduate, who floods this city with literature saying that he is the only dentist in the city who thoroughly sterilizes his instruments after each operation.—J. K. Conroy, in *Era*.

**Decrease in Registration.** The Dentists' Register, for 1906, contains the usual information as to registration, fees upon registration, the Dental Act in full and other important matters connected with the dental profession generally. The important notice to registered dentists which first appeared in the issue of 1904, again is prominently displayed on page 3, and it behoves all registered practitioners to see to it that their names are duly included in the list at the end of the book. It is interesting to note that the number of practitioners who were registered under the Act of 1878, as being before that date in *bona fide* practice of dentistry, is gradually though slowly shrinking, the percentage being 16 percent as against 18 percent in 1903 of the whole number of registered dentists.—*Dental Record.*

**Aconite and Chloroform.** Dr. Louis Jack, in a paper read before the New York Stomatological Society and printed in the *Journal*, says that after caping a pulp there is sometimes a tendency to pain in the immediate region, which is also often reflected to the ear, and a decided reaction to cold applications. In such cases, dry the gum tissue and rub away adhering mucous and apply on a pledget of cotton, for from fifteen to twenty seconds, a lotion made of one part chloroform and two parts of aconite. The action of the chloroform is to accelerate the absorption of the aconite, which depresses the sensory nerves and equalizes the blood pressure at the apical region. The chloroform also acts as a counter irritant primarily with a secondary sedative reaction. Such applications have an endurance of several hours, or in some cases, for one or two days.

**Decadent Teeth.** The editor of the *Sheffield Daily Telegraph* writes in that organ: "Since the Education Committee have mentioned the subject of the teeth of school children, we have been astonished to notice the almost desperate condition of the teeth of many of the young girls one can see going about the city. In holiday times, when one sees the city's youths and maidens more, so to speak, in bulk, it is not difficult to pick up rather startling evidence under this head. We have seen dozens of otherwise pretty faces absolutely ruined so far as appearance goes whenever the lips were parted. Perhaps it is more noticeable at holiday time because the girls laugh more and talk more, and thus display their dental wreckage in more than ordinary prominence.

But is there any local reason for the early decay of the teeth? We do not know, but it might be worth inquiry. Certain it is that good teeth are the first essential for a healthy digestion; that minus them the race must become dyspeptic, peevish, sour, decadent. The food that passes some of the teeth we saw yesterday and on Monday must be nearly as bad by the time mastication is finished as some of that which is alleged to emerge from the canning factories of Chicago." —*Dental Record.*

**Organization.** Too much praise cannot be given Dr. Arthur Black for the splendid work that he has done. We have all worked hard to bring out the splendid results that we see at this time, but if any one man is to receive praise it is Dr. Black, with his splendid idea of organization. His methodical way has made it easy for this society to come out and be the envy of all State societies, and to-day you hear from the East and West, and North and South of State societies that are striving to follow the pace set by this society; but it is a pace that is going to be pretty hard to keep up with, because every pace that Illinois sets no matter whether it is in dentistry, agriculture, commerce or in professional work, is a pace that is going to keep them working overtime to keep up with; and so we are proud that we have men that can organize like Dr. Black, and we are also proud that we have men who will follow men like Dr. Black and make the society the envy of the whole country.—D. M. Gallie, in *Review.*

**Pulp Cappings.** Dr. Louis Jack recommends the capping of pulps with a paste made of eugenol and pure calcined oxide of zinc. The cavity is first carefully cleansed of disintegrated tooth materials and then wiped out with a warm solution of hydroaphthol in alcohol, which is allowed to remain in the cavity long enough to sterilize the infiltrated dentine about the pulp exposure. Little cups of metal of proper form and size to be admitted easily to the cavity and large enough to fully embrace the exposure, are filled with the paste of eugenol and zinc and deftly placed over the exposure and allowed to remain until they set sufficiently to admit the introduction of a more durable cement covering.

The metal caps can be made by rolling aluminum to a very thin sheet and with various sizes of punches, such as can be had at hardware stores, cut out a number of disks which can be made concave by round ended instruments on some yielding material like lead.—The *Journal.*

**Impression Materials.** The following from a recent number of the *Chemist and Druggist,* may interest some of our readers: Beeswax is mixed with a limited proportion of hard paraffine (1 to 7) when use as a dental impression wax to give the mixture the property of separating easier. Paraffine wax cannot replace the beeswax entirely, or in large proportions, as it lacks the plastic properties which are needed in dental work. The dental modeling compound or composition used for impressions in place of wax is composed of stearing, gum dammar, or cowrie, and French chalk colored with carmine. The several varieties—hard, soft, etc.—are made by varying the quantity of stearine and chalk in proportion to the dammar or cowrie. A receipt is given in "Pharmaceutical Formulas" as follows:

| | |
|---|---|
| Stearin - - - - - | ʒvij. |
| Gum dammar - - - - | ʒxij. |
| French chalk - - - - | ʒxxij. |
| Carmine to color. | |

Melt the stearine and shake into it the gum dammar, previously powedred, then add the chalk tinted with the carmine and perfume with ol. geranii, ʒ½.

**Tempering Gold.** Z. F. Vaughn, of Los Angeles, Cal., is reported to have discovered a process of tempering gold and other metals.

By his method of tempering, it is claimed that the ductile metals are not only hardened, but their density and homogeneity are brought to such practical perfection that a cutting edge is given keener and more durable than that of steel, because of the microscopic fineness and smoothness imparted to it.

The inventor's experiments were devoted chiefly to the manufacture of surgical instruments of gold, his first production being a hypodermic needle of tempered gold. He has added to this all the instruments now in use in modern surgery. Prominent surgeons have given these instruments practical tests and in every case the results, it is said, were entirely satisfactory. For surgical purposes, the inventor claims that instruments of tempered gold are superior to those of steel, because of their non-corrosiveness and the ease with which they may be sterilized. The secret of the process appears to be in the solution used in tempering.

Under a microscope, a razor manufactured by Mr. Vaughn showed a much smoother edge than a steel razor, and a test proved that it held its edge longer.—*Stomatologist.*

**Inherited Obligation.** It's very human to feel that if you have done something that will benefit the world, you ought to benefit by it yourself, and that the world, that gets the benefit, should pay you, its benefactor. But, oh! How easy it is to forget all the little things the world has done for you without sending you a bill, especially those fellows in the world who are not in the world because they happen to he dead. Next time you start off to Washington to take out a patent on a method (if it's a thing, it's different), while you're riding in the cars work it all over again in your mind, and figure out how many steps there are in your new process; if you do this, likely enough you'll find there are at least ten, and that nine of them are not new at all, but were freely bequeathed to you by those Dead Fellows. Then count up how little you have paid out in royalties for other men's secrets, that they did not keep secret. And query to yourself whether or not you ever could have invented your little own invention if those Dead Fellows had patented every thing they thought of? Believe me, my friend, if you do all this, you'll never finish your journey.

You'll get off those cars at the next stop and sell the balance of your ticket; and you'll use the money to buy flowers for the graves of those Dead Fellows.—The Pessimist, in June *Items*.

**Jenkin's Porcelain.** There is no necessity to have a pin that would resist 150 pounds pressure, because there is no such pressure upon any individual tooth. The use of caps united with bands, or partial bands, is a matter of discretion. It is not possible to lay down a rule. There are instances where a complete band is of the greatest value, but those cases are extremely rare. There are many teeth coming under the observation of practitioners in the course of a year that had been lost solely because they were improperly banded. A root is coniform, and it is almost a mechanical impossibility to make a perfect band, and when a perfect band is made of gold, the material is less easily tolerated than platinum. There are cases where it is most injurious to allow the pulp to remain alive, and under modern conditions the sacrifice of a pulp is not a serious matter. With regard to the question as to gold injuring porcelain, the gold never does harm, but the flux which is used frequently causes the porcelain to crack. With reference to porcelain crowns, I have been considering for some years the making of a low fusing porcelain which would not have the defects of the old porcelain, and the porcelain he had produced, if melted upon the tooth of any manufacturer and the placed under the hammer, would be sound and uninjured when the tooth was broken to pieces. N. S. Jenkins, in *Brittish Dental Record.*

**Double Step Hood.** Under this title, Dr. T. P. Hinman, of Atlanta, describes in the July *Cosmos*, a method of attaching bridges to the cuspids and other incisal teeth by means of a plate attached to the lingual surface. The lingual surface is ground into the form of a double step, one step is made on a line between the mesial and distal angles and the other within a sixteenth of an inch of the gum margin. To this form, a plate of thin pure gold is carefully fitted, covering all of the lingual surface, and almost the entire distal surface, if the dummy to be attached is a bicuspid, or if a lateral incisor dummy is to be attached, it should extend over the mesial surface. This is to secure a larger area for soldering. Two pins are set into the backing on the upper step so that they enter the dentine in strong dentine on either side of the pulp. Another pin is set into the lower step. This is then flowed over with high grade solder which makes the backing ridid and capable of sustaining the stress of the bridge without change. The other abutments may be made with a shell crown, a gold inlay, a post in the root or by a similar step hood on another incisal tooth. When the bridge is completed it is cemented to place. The pins in the hood assist in anchoring the hood firmly to the teeth. The advantages claimed are that it is artistic; no bands are used to irritate the gums and peridental tissues; and it obviates the necessity of devitalizing the pulps as when a Richmond crown is used, or a post is set into the root canal. Dr. Hinman has used the method several years and pronounces it a useful and practical method.

**Delayed Extraction of Abscessed Teeth.**

Another trouble which presents for treatment, and which is, I regret to say, in many cases the result of poor treatment, is often characteristic of the malpractitioner. It is the treatment applied to abscessed teeth, where extraction has been delayed several days, after it has been determined that extraction is necessary.

I have heard men say that the extraction of an abscessed tooth, during that period when the face is swollen is a very dangerous procedure, and I dare say that it is not an uncommon practice even in this profession to advise postponement of the extraction until the tissue resumes a normal condition, or the swelling "goes down." I have never, however, been able to get a satisfactory explanation as to why the extraction should be delayed. This is inflammation again, and, as always, the removal of the cause should be the first step in its treatment. The pathological changes which have taken place in the tooth or surrounding tissues cause it to be a foreign body, and the system tries to expel it. The anatomical conditions which are associated with its position, however, prevent this from taking place, therefore, the tooth acts as an irritant. All the other stages of inflammation follow this step and we get destruction of the tissues. I will show you a case on the screen later where the destructive process involved the molar and maxillary bones, parts of which I removed to bring about a cure.

There is no more reason why an abscessed tooth should be retained in the jaw simply because the face is swollen, than there would be to allow a splinter of wood to remain in the hand, a piece of glass in the foot or a piece of steel in the eye, or for a gangrenous appendix to be left undisturbed until the inflammatory symptoms had subsided before attempting to remove it, since the forms of pathology are identical.

The fact that some cases of tooth extraction result in the death of the individual does not mean that the extraction of the tooth was the cause; such misadventure is usually due to the negligence of the operator in overlooking the very important steps of mouth sterilization before and after extraction. I base this statement upon clinical fact that most, if not all, deaths following tooth extraction are due to septic pneumonia, oı oral sepsis.—*Items of Interest.*

**An Equitable Fee Basis.** I would lay down this proposition—the only rational basis upon which to make our charges is *the burden imposed upon the operator.*

Each operator, of course, has some unit from which to work. I would say, find out what is about an average charge made for any particular kind of work by the better class of dentists in your community, then make that your starting point or unit. Suppose you find the average charge for a gold filling to be three dollars, then your fee unit is three dollars. Now, starting from this unit, build accordingly. From experience you will find that the average time consumed in filling the smaller cavities is, say, thirty minutes. This, then, is your work unit. Now some days you fill four cavities for one patient. There is not a great deal of difference in the time consumed on each. The first requires thirty minutes, and the fee unit is three dollars. The next one requires fifty minutes. Ordinarily we charge the same. But why should we donate to the patient twenty minutes of time which has a definite value? This extra twenty minutes means just that much more of a burden to you. The rational thing to do is to add proportionately to your unit fee of three dollars—that would make this one four dollars.

Is not this a simple, rational basis upon which to work? Do you do it? Do the same thing in computing charges for the other two. *Do the same for every patient, every time.*

In making two different artificial dentures use the same rule should hold good if you are a professional man—but if you are only a mechanic it will be proper to saw them off in five and ten dollar lengths.

Pursue this method in computing charges for all classes of your work, taking the simplest operation in each as your unit and adding to that in proportion as the burden increases.

Probably we err more in this direction in making amalgam fillings, in extracting teeth and in treating diseased teeth than in any other part of our work. And consequently we suffer more from it than we are aware.

In all these cases the rule calls for the most careful and conscientious adjustment in order that only justice to both operator and patient be done. As you grow in experience and reputation you should increase the value of your units. Make this change in method slowly until you can educate your patients to the fact that work means money to you.—J. D. Moody, in *Pacific Gazette.*

**A Sensible Comment on Dental Education.**

Dr. James Truman, in a paper read before the Seventh and Eighth District New York Dental Society, and published in the July *Cosmos*, makes the following timely comment of the much discussed subject of Dental education. We ought to print the entire article, but we can only give his concluding paragraph. He says: "What is most needed in this country by the dental profession is to stop distracting discontent with our dental schools and at our educational standards. We should follow the example of our English friends and pay more attention to our home market and less to the export trade—which, rest assured, has gone forever. Put the well-thumbed almanacs away on a high shelf, or, if you please, where the children or dogs will tear them up. Judge the dental colleges by what they have done for you and your professional neighbors, and by what their graduates have done to give the profession of our own land the high standing it has. There is room for improvement, unquestionably, if the profession will only provide the means. Make it worth while for a talented dentist to accept a professorship in a dental college, and stop the serious loss which the profession sustains when promising teachers, because underpaid, are compelled to quit teaching when their practices become exacting and remunerative. Teach the public to respect your calling by respecting it yourselves. Do not demean yourselves by proclaiming the shortcomings of the school from which you graduated, but lend some encouragement. There is far too much berating the colleges done in the dental societies. The colleges are supplying plenty of good professional timber, if it shall be properly handled by the profession. Instead the newly graduated are led to think that their teachers and institutions are not what they supposed they were. Trivial matters are magnified, instead of being overlooked. Change the tune. Teach the young men that their teachers, their colleges and their curriculums are worthy, and when things seem to be wrong the trouble may be in themselves, as it too often is. Let the craze for higher standards rest a while. Quit squabling and get to work, making the professional organization what they can and should be. Make it unprofitable for any man not to be an active member of some one. Teach young men that our profession is an honorable one and worthy members will always command the respect of the community. This is what we need most now."

**A Bragging Dental Society.** We reprint the following extract from the transactions of the Illinois Dental Society as published in the July *Dental Review*, not to criticise, but to commend and to help spread the idea of organization and enthusiasm in association work as a means of professional advancement:

Dr. C. B. Rohland, our president, congratulated himself upon presiding over the very largest and very best society in the world. That has been the song of the members of that society ever since I have been a member of it, so that it has become a habit. When do you suppose that habit began? Who among all the members here would you say that in proportion to his scholarship and education was the most modest man on the floor? I don't mean myself, now (laughter). Who among all the members here would you naturally suggest as being one of the most modest of men in proportion to his scholarship, a man we all love? (A voice, Dr. G. V. Black.) Certainly. This is the way it began. Somewhere back in the 70s Dr. Black was president of the society and on retiring from the chair he found occasion to make some remarks, and he thanked them for the honor conferred, and said he felt it to be an especial honor to preside over the largest and best dental society in the world, and from that date it passed into a habit. At that time the society had barely one hundred members, and the average attendance was about thirty or forty, I am quite sure not above that at that time, and yet he speaks of it as the largest and best dental society in the world. What shall we say about it when we have over thirteen hundred members, largely due to the son of the man who made that braggadocio remark—what shall we say of the society now, with over thirteen hundred members and an average attendance of probably five hundred, which we fully expect, and which we had last year? If we had the big head then where will we be now? That is a good habit, however, to have, and don't you know I think the success of this society is attributed largely to that spirit, for in all the thirty-two years I have been a member of this society it has been the habit of members to speak of thier society as the very best society going. They always bragged about it, and we finally talked about it so much that we made others believe we were the best society going, and we got the reputation of being not only the best society, but I am afraid the most conceited society too, because I remember George Edwin Hunt on the floor of this society told us that we were the most conceited society and there wasn't one that had the sand to get up and dispute it. (Laughter.) It is largely due to such conceit that this society has done the good work it has. It is like a child. If a parent makes the child believe that he is the best and most obedient child around, that child is going to live up to that reputation. On the other hand if he says "I can't do anything with that kid at all," the child will live up to that reputation. We have given ourselves a reputation and we must live up to it. It is up to those who are now joining us to see that we do make this the very best society in the world. We have the people, we have the ability and we have the energy.

I wanted to draw attention to the gentleman from whom all this spirit of brag started—the very last man in the society that you would think of, Professor G. V. Black.

Dr. G. V. Black: Mr. President, I don't feel like making any apology for making that remark attributed to me. I believe it was a fact then—no brag about it. It has been a fact ever since, and I hope these young men will keep it a fact right along down the years—the largest State society in the world, and the best; the best fellowship, the best of love one for another, and the most regular attendance upon the meetings of any society. It is the attendance upon the meetings that developes young men and makes them grow. I don't think I would have been worth two cents if it had not been for this society—certainly not to the world and not to dentistry.

I have attended nearly all the meetings. My first affiliation was with the St. Louis society, before this society was organized, but when this society began to show its work I came in with it and have been with it ever since. The first meeting that I missed afterward I believe I had to stay at home to meet that boy (pointing to Arthur D. Black.

Dr. Geo. Cook: He is bragging again.

Dr. Black: Well he insisted on keeping me away from the meeting, and I did not like that at that time, not a bit. I don't know that this matter of bragging hurts us any if it is done modestly. (Laughter.)

**Oxidizing Agents Employed in Dentifrice.** The desirability of the addition of an actively oxidizing substance to a dentifrice composition is yet an open question.

The substances employed to yield oxygen must, almost of necessity, be of an unstable character, so that their decomposition, with accompanying liberation of oxygen, may be effected without the need of applying heat, or the employment of strong acids. This limits our selection to such chemical substances as undergo decomposition in the presence of moisture, or organic matters, or (usually) both.

To incorporate such agents into mouth washes, pastes or even powders, not kept hermetically sealed, is in most instances of questionable value, for in a very short time, even measurable in hours, the oxygen they liberate on contact with moisture is lost, and the dentifrice only holds an inert substance. Even the action of atmospheric moisture will, in a short time, occasion the loss of activity of the oxidizant in corporated in a tooth powder.

Assuming, however, that the oxidizing agent preserves its entity in a dentifrice, then its continued use may not be altogether free from danger, as the active oxidation it occasions, always attended by the production of heat, from chemical action, may be the cause of serious harm.

Many of these oxidizing substances are of a caustic nature and as such are unfitted for indiscriminate use by the laity.

The fact that no harmful effects appear from the use of a dentifrice to which considerable quantities of an actively oxidizing agent have been added is to be explained, in many instances, in the decomposition of the oxidizant at the time of, or shortly after, its incorporation, so that the only oxidizing feature of the dentifrice is found in its name.

To such dental practitioners, then, as contemplate adding an oxidizing agent to a dentifrice composition, our advice is that of "Josh Billings" to the poor young man contemplating matrimony—"Dont."

The proper employment of the efficient chemical substance yielding oxygen when undergoing decomposition, is in the office and under the administration of the dental practitioner.

We have found that we may employ only such oxidizants as will undergo decomposition when in contact with moisture, organic matter, or very weak acid.

The oxygen liberated at the desired place will be in its most active form, the form known to the chemist as the "nascent" state, and the dentist must not forget that while in such active condition oxygen will attack metal filling (other than gold) in teeth, and occasion considerable pain if in contact with carious spots.

The oxydizing agent that has proven most serviceable to the dentist is the familiar peroxide of hydrogen ($H_2O_2$) or aqua hydrogenii dioxidi, U.S.P.

The solution of hydrogen dioxide is a slightly acid, watery preparation containing 3 percent by weight of true hydrogen dioxide, or capable of giving rise, in its decomposition, to ten times its own measured volume of oxygen, *i. e.*, a pint of official hydrogen dioxide solution should, on decomposition, give rise to ten pints of nascent oxygen.

According to the Pharmacopia of 1890 this substance is prepared by thoroughly mixing barium dioxide with distilled water (keeping the temperature below 50 degrees F.), then adding dilute phosphoric acid (1 part to 3 water), shaking and filtering, and to the clear filtrate adding dilute sulphuric acid, by drops, as long as the addition of the latter causes a cloudy appearance, then mixing this hazy liquid with starch and filtering, when the resultant clear solution is assayed and its strength, by dilution or concentration, is brought to the standard 3 percent.

A number of preparations having an allied action with $H_2O_2$ are now on the market; among them we notice:

Hydrozone. This is a solution of peroxide of hydrogen made by Marchand, and is of three times the strength of the official preparation, containing 9 percent of the $H_2O_2$ and liberating 30 volumes of nascent oxygen.

Acetozone $(C_6H_5CO\text{-}COCH_3)\ O_2$: This substance occurs in crystal form and dissolving in from 1,000 to 10,000 parts of water yields a solution that readily parts with oxygen.

It is sometimes employed in powdered form, when through its slow solution in the moisture of the tissues, it produces a continuous supply of oxygen.

Glycozone is obtained by treating glycerine with fifteen times its volume of ozone.

Peroxide of sodium, $Na_2O_2$. This substance occurs as a yellowish white powder. It is prepared by placing slices of the metal sodium upon an aluminum tray and causing the tray so laden to be drawn through a long metal tunnel through which air freed from $CO_2$ and heated to 400 degrees C. is forced in a direction opposite to that in which the aluminum carrier is traveling.

When sodium dioxide is brought in contact with moisture it is converted into sodium hydrate and forms peroxide of hydrogen as: $Na_2O_2+H_2O_2={}_2NaOH+H_2O_2$, so that in its use we gain the advantage of a freshly produced peroxide of hydrogen of concentrated character.

Calcium dioxide, $Ca_2O$. This substance may be prepared as a white powder, by adding peroxide of hydrogen to lime water. It is manufactured by mixing solutions of peroxide of sodium and calcium chloride. It is, unless carefully prepared, some what caustic in its action.

The availability of the metallic perborates as non-irritating, neutral or alkaline oxidizing substances has recently been called to the attention of the dental profession.

Of the perborates, two at least, the sodium and the magnesium salts, offer in their freedom from acidity and non-irritating character, almost ideal agents for incorporation in dentifrice composition, if this be thought desirable.

They, sodium perborate and magnesium perborate, are stable, not suffering change from moisture in air, and when in solution or paste appear to still exert an appreciable oxidizing function.—Dr. H. H. Boom, in *Stomatologist*

